# Supplementary figures and images for: Molecular and epidemiological analysis of a Burkholderia cepacia sepsis outbreak from a tertiary care hospital in Bangladesh
Source: PLoS Negl Trop Dis. 2020 Apr 9;14(4):e0008200. doi: 10.1371/journal.pntd.0008200 (PMC7173934; doi:10.1371/journal.pntd.0008200)

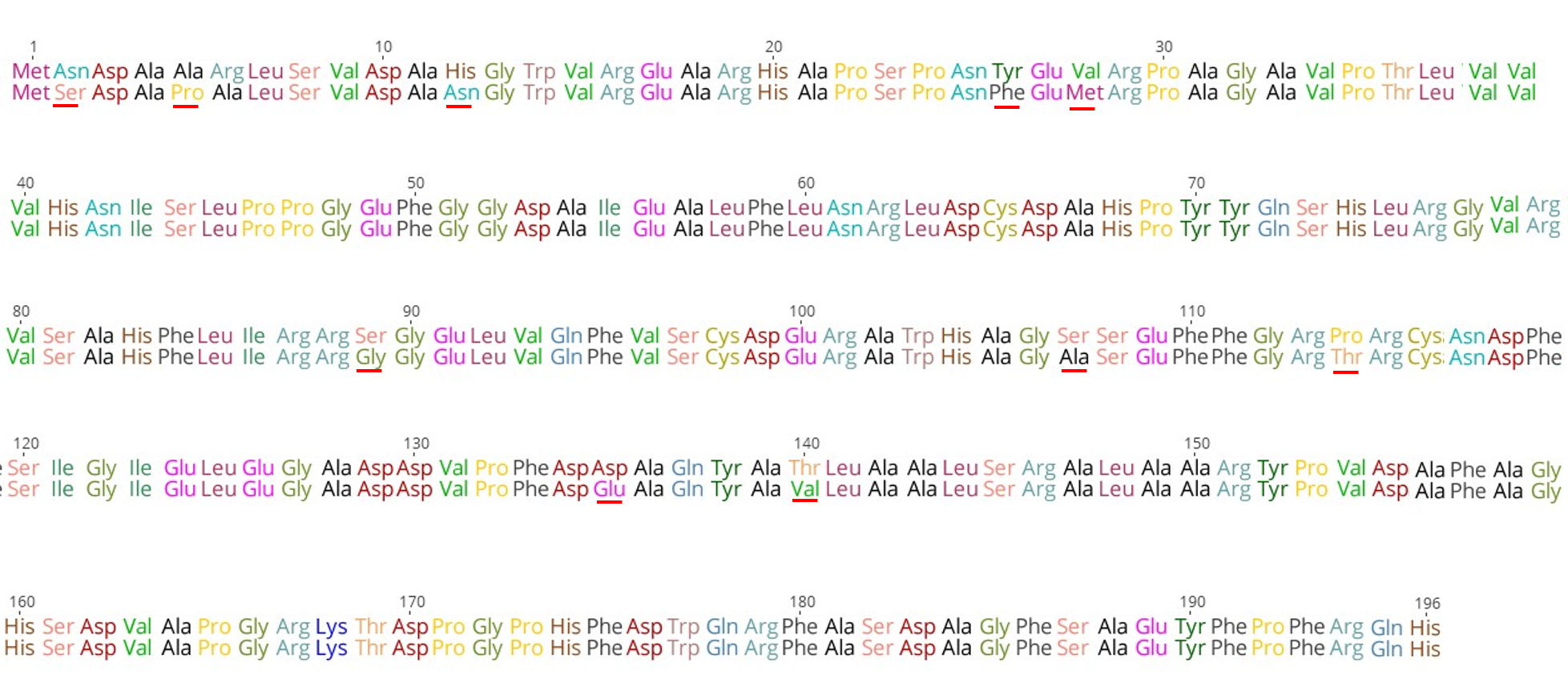

Supplement: S1 Fig — Substitutions are underlined by red. (TIF) [file pntd.0008200.s001.tif]
