# Supplementary material for: Molecular and epidemiological analysis of a Burkholderia cepacia sepsis outbreak from a tertiary care hospital in Bangladesh
Source: PLoS Negl Trop Dis. 2020 Apr 9;14(4):e0008200. doi: 10.1371/journal.pntd.0008200 (PMC7173934; doi:10.1371/journal.pntd.0008200)
Supplement: Attachment — Submitted filename: Comments.docx [file pntd.0008200.s003.docx]

General comments:

The manuscript is scientifically sound and the results are clearly demonstrated.

Specific comments:

Abstract: Why the outbreak cases were confined to the burn critical care unit or the paediatrics department? Usually, in hospital of our country, we would insulate suspicious infectious patients in isolated wards, like department of infectious diseases or Intensive care unit, not the burn critical care unit or the paediatrics department. An infectious outbreak in a burn unit would be considered as an urgent health accidence.

Line 52: “Antibiotics are used empirically to manage infections and invariably offered to all patients admitted.” So, what is the empirical antibiotic regime to all patients admitted. I cannot see the complete information of table 1 in the PDF manuscript. Generally, the antibiotics used should be variable to individuals based on the different situations, not the same.

Line 122: “Virulence factors (VFs) were evaluated by VFDB [19].” Please clarify “VFDB” in detail, as well for other short names in Methods.

Line 132: “We mapped single nucleotide polymorphisms (SNPs) of all genomes including global B. cepacia from database (n=105) to the first isolated strain in our study.” Here, are “all genomes” include B. cepacia (n=15) isolated in this study and B. cepacia from database (n=105) ? 120 isolates?

Line 137: About Investigating possible outbreaks. Have you tried to isolate possible pathogens from contaminated things in wards of the hospital or the places those patients lived? Can you describe the details about the clinical investigation, other than the molecular epidemiological analysis?

Line 179: B. cepacia isolated in this study were assigned as a single novel ST, ST1578 (Fig 3). It is interesting that there is only a single known ST. Are there any B. cepacia strains isolated from other sample types other than blood?

Line 180: All strains were grouped into 9 clusters (Fig 3). Here, all strains are 120 isolates? Or 106 cases?

Line 236: “Apart from two strains,…” changed to “Apart from this two strains,…”

Figure 5: The resolution is not high enough and may influence its readability.
