# Supplementary material for: Molecular and epidemiological analysis of a Burkholderia cepacia sepsis outbreak from a tertiary care hospital in Bangladesh
Source: PLoS Negl Trop Dis. 2020 Apr 9;14(4):e0008200. doi: 10.1371/journal.pntd.0008200 (PMC7173934; doi:10.1371/journal.pntd.0008200)
Supplement: Attachment — Submitted filename: rebuttal.docx [file pntd.0008200.s004.docx]

**Point-point Rebuttal**

**General comments:**

The manuscript is scientifically sound, and the results are clearly demonstrated.

Thank you for considering our manuscript for revision.

**Specific comments:**

1. Abstract: Why the outbreak cases were confined to the burn critical care unit or the paediatrics department? Usually, in hospital of our country, we would insulate suspicious infectious patients in isolated wards, like department of infectious diseases or Intensive care unit, not the burn critical care unit or the paediatrics department. An infectious outbreak in a burn unit would be considered as an urgent health accidence.

Regrettably, we detected this outbreak at Dhaka Medical College Hospital (DMCH), retrospectively, after receiving the strains at Cardiff University. DMCH is the largest public hospital in Bangladesh, providing health care facilities 3-4 times its bed capacity. There is no isolation ward for suspicious, or even confirmed, infectious patients. Antibiotics have been prescribed empirically at the hospital setting and routine culture and sensitivity testing has only been done sporadically. Hence, the outbreaks are rarely identified and less so managed.

2. Line 52: “Antibiotics are used empirically to manage infections and invariably offered to all patients admitted.” So, what is the empirical antibiotic regime to all patients admitted. I cannot see the complete information of table 1 in the PDF manuscript. Generally, the antibiotics used should be variable to individuals based on the different situations, not the same.

There is no guideline for empirical antibiotic prescription at DMCH. Mostly antibiotics have been prescribed based on availability of hospital supply (personal communication). Our dataset suggests that the most common antibiotic usage at DMCH is ceftriaxone and at the burns unit, a combination of ceftriaxone and an aminoglycoside is common. It is obvious for our data that patients infected with *B. cepacia* are mostly managed with aminoglycosides, but the bacteria are intrinsically resistant to aminoglycosides. In column 13 of Table 1, we included the name of antibiotics prescribed to the patients during their hospital stay. As a part of our pilot study at DMCH, we collected blood specimens from patients with >20% admitted to the burns’ ICU/HDU, but the clinical reporting and patient management was fully carried out by DMC clinical microbiology team and hospital physicians. For the purpose of this study, we only collected positive cultures as per the granted permission of the DMCH ethical board.

We apologize that our presentation in this regard is unclear. To clarify, we have amended line 54 to line 58 (please see below):

‘*Herein, we report a B. cepacia outbreak from a burn unit for a protracted period and the outbreak cases were found to be managed by inappropriate antibiotic therapy. B. cepacia is a Gram-negative bacterium, mostly a lung pathogen for cystic fibrosis patients and also produce disease in immunosuppressed patients*.’

3. Line 122: “Virulence factors (VFs) were evaluated by VFDB [19].” Please clarify “VFDB” in detail, as well for other short names in Methods.

Line 122 has been amended as suggested. Other short forms in the manuscript are now added as full form (please see below):

‘Line 122: Virulence factors (VFs) were evaluated by virulence factor database (VFDB) [19].

Line 103: Matrix-Assisted Laser Desorption/Ionization-Time of Flight (MALDI-TOF) mass spectrometry

Line 109: clinical and laboratory standards institute (CLSI)

Line 117: Burrows-Wheeler aligner (BWA)

Line 120: comprehensive antibiotic resistance database (CARD)

Line 138: interactive tree of life (iTOL)’

4. Line 132: “We mapped single nucleotide polymorphisms (SNPs) of all genomes including global B. cepacia from database (n=105) to the first isolated strain in our study.” Here, are “all genomes” include B. cepacia (n=15) isolated in this study and B. cepacia from database (n=105)? 120 isolates?

In total, *B. cepacia* will be 106. Apologies for the mistake. To avoid confusion, the line has been amended (please see below):

‘*We mapped single nucleotide polymorphisms (SNPs) of global B. cepacia from database (n=91) and our outbreak strains (n=15) with the first isolated strain in our study, dm93 (used as reference genome) using Snippy (3.2) [21].*’

5. Line 137: About Investigating possible outbreaks. Have you tried to isolate possible pathogens from contaminated things in wards of the hospital or the places those patients lived? Can you describe the details about the clinical investigation, other than the molecular epidemiological analysis?

As we already discussed that we identified the outbreak retrospectively, we suspect the outbreak was sustained for a long period due to hospital environmental contamination, however we did not evaluate linkage between the hospital environment and the patients’ infection in this study which we mentioned in discussion in Line 309:

‘*Although we did not fully evaluate the epidemiological link between patients and the environment, epidemiological and molecular data suggest that the outbreak clone was circulating within DMCH over a protracted period (Fig 2)*.’

6. Line 179: *B. cepacia* isolated in this study were assigned as a single novel ST, ST1578 (Fig 3). It is interesting that there is only a single known ST. Are there any *B. cepacia* strains isolated from other sample types other than blood?

In our study, *B. cepacia* was isolated only from blood and all were assigned as single clone.

7. Line 180: All strains were grouped into 9 clusters (Fig 3). Here, all strains are 120 isolates? Or 106 cases?

In total, there are 106 strains. We have amended line 180 (please see below):

All strains (n=106) were grouped into 9 clusters (Fig 3).

8. Line 236: “Apart from two strains,…” changed to “Apart from this two strains,…”

We have amended the sentence as suggested (Line 237)

9. Figure 5: The resolution is not high enough and may influence its readability.

Figure 5 has been adjusted accordingly with the revision.

**Other amendments:**

1. Table 1 and Table 2 have been formatted to fit in the word.
2. Accession numbers have been included at the end of method section (Line 149-Line 163)
