# Supplementary material for: Molecular and epidemiological analysis of a Burkholderia cepacia sepsis outbreak from a tertiary care hospital in Bangladesh
Source: PLoS Negl Trop Dis. 2020 Apr 9;14(4):e0008200. doi: 10.1371/journal.pntd.0008200 (PMC7173934; doi:10.1371/journal.pntd.0008200)
Supplement: Attachment — Submitted filename: rebuttal-31-01-20.docx [file pntd.0008200.s005.docx]

**Point-point Rebuttal**

Reviewer #1: More detailed information may be need for the definition of the outbreak. Considering the DMCH is a tertiary care hospital, the Infection Control Division would have taken actions for better disinfection and clinical investigation, rather than observing the hospital stay over 11 months. Such a long time for a response to nosocomial infection emergency. Authors should provide more detailed information about clinical investigation.

We have undertaken these studies to provide prima facie evidence for enhanced IPC programs. We have worked in the DMCH for several years and have encouraged the hospital and the DGH to invest in IPC. The current study affirms our previous findings that greater awareness and investment is needed for IPC programs but regrettably resources are limited to implement such a program. However, it is hoped that studies such as these will be persuaded to the Bangladeshi government to see the benefit of strong IPC programs.

Please see the modified text for further detail. We have amended the followings:

Line 55-59: ***Herein, we report a B. cepacia outbreak from a burn unit for a protracted period where the patients were treated with inappropriate antibiotic therapies. B. cepacia is a Gram-negative bacterium, mostly a lung pathogen in cystic fibrosis patients but can also produce infections in immunosuppressed patients.***

Line 95-96: ***Detailed microbiological analysis of isolates, including Whole Genome Sequencing (WGS), performed at Cardiff University, were undertaken retrospectively.***

Line 100-103: ***Clinical data was collected following positive culture results from the local laboratory. Additional clinical follow up information was not available in the current study, except for outcome (discharge, death, discharge against medical advice {DAMA}).***

Reviewer #3: Acceptable.

Reviewer #1: Burkholderia cepacia sepsis outbreak among human is rare and neglected in clinic. The results basically match the analysis plan and the evidence is sufficient for the definition of the outbreak from the perspective of molecular epidemiological technology. The results are clearly demonstrated, and the figures are of sufficient quality. If possible, more background information of clinical investigation is better to predict the epidemiological links between cases. The gap between resistance gene prediction and clinical antimicrobial phenotype is often obvious.

We appreciate the importance of blending rich clinical and demographic information with molecular characterisation and fully agree with your comments. Regrettably, at the DMCH where patient information is not digitalised, it is challenging to obtain additional patient information but nonetheless we have attempted to justify the limitations of our study more clearly in the current draft of the manuscript.

Line 148-152: ***Detailed epidemiological information and subsequent infection control measures were not available as the microbiological information was only available retrospectively. Infection Prevention and Control (IPC) measures were not implemented at the time due to the limited and untimely microbiological information at the DMCH.***

Reviewer #3: -Are the results clearly and completely presented?

No.

If we look at the table 1, an important finding is there that those patients who were given empirical antibiotic either levofloxacin or meropenem were discharged (though no. is small) and none of them died. Authors need to look at the susceptibility of these antibiotics in the given patient. Accordingly, this information may be included in the manuscript.

Another observation is that in case 1 & 3, despite on other antibiotics, patients get discharged. Did these patients have clinical signs of sepsis or do these cases fall under pseudobacteremia for which B. cepacia complex is well known?

Thank you for these comments and suggestions. As reasoned above, we are somewhat limited by the quality of the clinical information available to us. In this regard, the changing of antibiotic regimes is not always recorded nor on patient discharge. The perceived anomaly may be partly explained by the interpretation of *B. cepacia* breakpoints– EUCAST do not provide breakpoints for many antibiotics. CLSI however do, but we understand discussions are ongoing between EUCAST and CLSI which could lead these being changed in the near future. Therefore, whilst we think the high MICs observed for many agents indicate they are unlikely to be therapeutically useful. We have discussed these limitations in the main text, especially in the discussion section.

Line 293-303: ***A limitation of the current study was the lacked sufficient clinical information to access and to investigate why the mortality rate was significantly lower for B. cepacia cases. At the time of enrolment many of these patients were on “inadequate” antibiotics based on our susceptibility data. However, there are limitations to the interpretation of AST results for B. cepacia and we lack data on whether antibiotics were changed later during hospital stay. It is possible there were other factors favouring survival in the group infected with B. cepacia, including possibly relatively lower pathogenicity of B. cepacia as a cause of bacteraemia. B. cepacia has been described as a cause of pseudo-outbreaks [32]. Although this possibility should be considered here the timing of infections suggest this was not the case, and a persistent environmental source of cross contamination on the burn unit was a more likely cause.***

Reviewer #1: Generally, BC is not common pathogen in burn care unit. This is an out-of-control emergency of nosocomial infection. So do not mention too much about the universality of BC infection in burn ICU but take a deep research about the definition of the criminal bacteria and the links between cases.

The current paper has some limitations regarding obtaining very details clinical information and lack environmental correlation with the clinical cases. To improve IPC in the Bangladeshi public hospitals, we have started working with Bangladeshi government and trying to understand the evolution of pathogens causing clinical infections in the hospital settings with poor IPC and to link between clinical infections and environmental contamination. This findings in the current paper is mainly the evidence to inform the importance to work further details in this area by the Bangladeshi government/researchers.

Reviewer #3: -Are the conclusions supported by the data presented?

No.

Conclusions: Environmental contamination is a potential source of outbreak in a hospital setting with very poor infection control policies.

The manuscript lacks the data to support this conclusion. Only the isolates from burn ward have been collected without even mentioning about the possible source.

Despite pointing out earlier, it has still not been addressed as a limitation in the manuscript.

Thank you for these comments and we acknowledge this limitation. Regrettably, we do not have additional information on the burn’s environment or the swabbing of staff and equipment to correlate the source of contamination/infection. However, we have modified the conclusion to address these concerns. Notwithstanding, B. cepacia can persist in the environment and the fact that there were intermittent infection cases of a closely related/clonal B. cepacia isolates over a prolonged period of time, it is not unreasonable to speculate that the bacterium has niches on the unit where it can persistently colonise and infect. Please see the text in Discussion where we have addressed this issue.

Line 46-49: ***Environmental surveillance is required to investigate the aetiology and mode of transmission of the B. cepacia outbreak. Systematic management of nosocomial outbreaks, particularly in resource limited regions will mitigate transmission and improve patients’ outcomes.***
